# Novel association of five HLA alleles with HIV-1 progression in Spanish long-term non progressor patients

**DOI:** 10.1371/journal.pone.0220459

**Published:** 2019-08-08

**Authors:** Eva Ramírez de Arellano, Francisco Díez-Fuertes, Francisco Aguilar, Humberto Erick de la Torre Tarazona, Susana Sánchez-Lara, Yolanda Lao, José Luis Vicario, Felipe García, Juan González-Garcia, Federico Pulido, Félix Gutierrez-Rodero, Santiago Moreno, Jose Antonio Iribarren, Pompeyo Viciana, Carlos Vilches, Manuel Ramos, Laura Capa, José Alcamí, Margarita Del Val

**Affiliations:** 1 National Center for Microbiology, Instituto de Salud Carlos III, Majadahonda, Madrid, Spain; 2 Infectious Diseases Unit, IBIDAPS, HIVACAT, Hospital Clínic, University of Barcelona, Barcelona, Spain; 3 Viral Immunology, Centro de Biología Molecular Severo Ochoa (CSIC-UAM), Madrid, Spain; 4 Departamento de Histocompatibilidad, Centro de Transfusión de Madrid, Madrid, Spain; 5 Hospital Universitario La Paz, Madrid, Spain; 6 HIV Unit, Instituto de Investigación Hospital 12 de Octubre (i+12), Madrid, Spain; 7 Servicio de Medicina Interna, Unidad de Enfermedades Infecciosas, Hospital General Universitario de Elche, Alicante, Spain; 8 Infectious Diseases, Hospital Ramón y Cajal, Madrid, Spain; 9 Hospital Universitario de Donostia, San Sebastián, Spain; 10 Laboratory of Immunovirology, Biomedicine Institute of Sevilla, Virgen del Rocío University Hospital, Clinic Unit of Infectious Diseases, Microbiology and Preventive Medicine, IBIS/CSIC/SAS/University of Sevilla, Sevilla, Spain; 11 Inmunogenética e Histocompatibilidad, Instituto de Investigación Sanitaria Puerta de Hierro, Majadahonda, Madrid, Spain; University of Texas Rio Grande Valley, UNITED STATES

## Abstract

Certain host genetic variants, especially in the human leucocyte antigen (HLA) region, are associated with different progression of HIV-1-induced diseases and AIDS. Long term non progressors (LTNP) represent only the 2% of infected patients but are especially relevant because of their efficient HIV control. In this work we present a global analysis of genetic data in the large national multicenter cohort of Spanish LTNP, which is compared with seronegative individuals and HIV-positive patients. We have analyzed whether several single-nucleotide polymorphisms (SNPs) including in key genes and certain HLA-A and B alleles could be associated with a specific HIV phenotype. A total of 846 individuals, 398 HIV-1-positive patients (213 typical progressors, 55 AIDS patients, and 130 LTNPs) and 448 HIV-negative controls, were genotyped for 15 polymorphisms and HLA-A and B alleles. Significant differences in the allele frequencies among the studied populations identified 16 LTNP-associated genetic factors, 5 of which were defined for the first time as related to LTNP phenotype: the protective effect of HLA-B39, and the detrimental impact of HLA-B18, -A24, -B08 and –A29. The remaining eleven polymorphisms confirmed previous publications, including the protective alleles HLA-B57, rs2395029 (HCP5), HLA bw4 homozygosity, HLA-B52, HLA-B27, CCR2 V64I, rs9264942 (HLA-C) and HLA-A03; and the risk allele HLA bw6 homozygosity. Notably, individual Spanish HIV-negative individuals had an average of 0.12 protective HLA alleles and SNPs, compared with an average of 1.43 protective alleles per LTNP patient, strongly suggesting positive selection of LTNP. Finally, stratification of LTNP according to viral load showed a proportional relationship between the frequency of protective alleles with control of viral load. Interestingly, no differences in the frequency of protection/risk polymorphisms were found between elite controllers and LTNPs maintaining viral loads <2.000 copies/mL throughout the follow-up.

## Introduction

The host genetic determinants influencing progression of HIV infection to disease and acquired immunodeficiency syndrome (AIDS) have been extensively studied in several cohorts of LTNP individuals of Caucasian ancestry. This is the case of several allelic variants in genes encoding the HIV-1 co-receptors and their ligands, such as CCR2 and CCR5, certain cytokines such as IL10, co-factors and interferon-induced proteins [1–9]. Among these host factors, the human major histocompatibility HLA class I complex has the strongest influence on HIV-1 progression. Thus, the HLA-B*57 and HLA-B*27 alleles are strongly associated with delayed HIV disease progression [10, 11] whereas HLA-B*35 is associated with accelerated progression to AIDS [12, 13]. In addition, control of viremia and protection from AIDS is associated with HLA bw4 allelic grouping homozygosity [14]. More recent studies identified allelic variants associated with control of HIV-1 replication in *HLA-C* and HLA complex P5 (*HCP5*) [15, 16], which in turn is in tight linkage disequilibrium with the HLA-B*5701 allele [17]. Studies based on genome-wide association strategies identified novel genetic variants associated with delayed disease progression [18–25], most of them within the HLA complex [19, 23, 24].

These data suggest that disease progression and HIV-1 replication is controlled by several loci of the human genome. However, known genes affecting disease progression and their variants do not fully explain the highly variable course of HIV-1 infection or its pathogenic mechanisms. The aim of the present study is to characterize genetically the large Spanish HIV LTNP cohort and to identify novel associations with disease control, employing a multicenter cohort of 398 Spanish HIV-1 positive patients compared with a control population of 448 healthy Spaniards. By comparing the genotype distribution of several SNPs as well as the frequency of HLA-A and HLA-B alleles, the present work proposes 5 novel HLA class I alleles related to maintenance of the LTNP status [defined as HIV-infected patients that maintain CD4-lymphocytic counts above 500 cells/uL for at least ten years in the absence of antiretroviral treatment (ART). Viral load is usually low in this group of patients (<10.000 RNA copies/ml, as defined in the Spanish LTNP-Cohort)]and confirms the role of known genetic markers associated with control of HIV-1 replication. The analysis of these genetic traits stratified by different phenotypes within LTNP patients, showed a differential effect according to the LTNP subcategory, evidencing the necessity to clearly define the LTNP condition in case/control association studies. In addition to supporting the category of EC with undetectable viral load (VL), we propose the use of a regularly maintained VL below the limit of 2,000 copies/mL as a new marker of profound and stable LTNP status.

## Materials and methods

### Patient samples

A total of 448 healthy bone marrow donors (HD), as well as 398 HIV-1 infected patients, comprising 55 AIDS patients, 213 typical progressors (TP) and 130 LTNP, were included in the study. The uninfected individuals were healthy Spanish donors from the Blood Transfusion Centre of the Community of Madrid, Spain, and are representative of the Spanish population [26]. All HIV-1 infected patients belonged to different cohorts of patients with samples stored at the HIV BioBank (Gregorio Marañón University Hospital, Madrid, Spain), which is integrated in the Spanish AIDS research network (RIS) [27] All the samples were collected from 2004 to 2007. CoRIS, the RIS cohort of adults with HIV infection, was launched in 2004 [28]. CoRIS is an open multicenter cohort of patients that are over 13 years of age and newly diagnosed with HIV infection in the participating hospital or treatment center they attend for the first time, and that are naïve to antiretroviral treatment. This study was reviewed and approved by the institutional Ethics committee for research and clinical trials" (CEIC) from Instituto de Salud Carlos III. All patients signed and informed consent to include their blood samples for scientific research including genetic studies in the Biobank of the Spanish AIDS Research Network. The information is subject to internal quality controls; once every 2 years, information on 10% of the cohort is audited by an external agency.

A total of 55 AIDS and 213 TP patients come from CoRIS. The AIDS group includes naïve patients late diagnosed after attending a participating center for the first time; the TPs are HIV-1 infected patients with CD4^+^ cell loss between 50–100 cells/μl per year. The 130 LTNP patients belong to the Spanish Cohort of LTNP (LTNP-RIS), a cohort similarly managed as above, and were naïve patients who have CD4^+^ T cell counts over 500/μl and VL < 10,000 copies/mL without antiretroviral treatment for at least 10 years after HIV diagnosis. The prototypical recruited HIV-1 infected individuals were male intravenous drug users of Spanish origin (Table 1).

**Table 1:** Summary of epidemiological characteristics of HIV-1 infected patients included in the analysis.

Characteristic		TP (n = 213)	AIDS (n = 55)	LTNP (n = 130)
Age upon admission, mean (min-max)		39 (17–69)	42 (22–61)	48 (30–76)
	Unknown, n			27
Country of origin, %	Spain	100	100	77.0
	Unknown			23.0
Sex, %	Male	69.0	74.5	53.8
	Female	31.0	25.5	26.2
	Unknown			20.0
Risk group, %	Intravenous drug user	68.1	60.0	64.6
	Homosexual/bisexual	9.4	14.5	5.4
	Heterosexual	16.4	16.4	13.1
	Others (transfusion, etc.)	3.3	7.3	2.3
	Unknown	2.8	1.8	14.6

Based on specific clinical data, including VL and time after seroconversion, we defined several LTNP subcategories. Thus, three mutually exclusive subcategories of LTNP have been analyzed, including ExLTNP, who are patients that lost LTNP status after at least 10 years after HIV-1 diagnosis; viremic non-controller LTNP (LTNP-N), who are LTNP maintaining detectable VL > 50 up to 10,000 copies/mL throughout the follow-up; and EC, defined as HIV-1 infected individuals with undetectable VL during follow-up. In addition, LTNP-C controllers includes a subgroup of LTNP-N maintaining VL <2,000 copies/mL throughout the follow-up; this subcategory includes all EC but also those LTNP-N with low VL. Blood samples were processed following standard procedures [29] and frozen immediately after their processing. Peripheral blood mononuclear cells were obtained from blood of all subjects included in the study and DNA was extracted.

### Sample genotyping

Genomic DNA was used for genotyping. Most SNP tested were typed using TaqMan SNP genotyping assay following manufacturer’s procedures and standardized protocols (Applied Biosystems), except for rs333 (*CCR5*-Δ32) and rs1801157 (SDF-1), which were determined by real time PCR employing the primers and probes described in S1 Table. The TaqMan Universal PCR Master Mix and standard thermocycling conditions were employed for all polymorphisms on an ABI PRISM 7000 system, and allele calling was performed using AutoCaller SDS Software v 1.2.3. (Applied Biosystems).

### HLA typing

Two-digit HLA-A and HLA-B typing was carried out using sequence-specific oligonucleotide (SSO) hybridization following manufacturer’s procedure and standardized protocol (RELI SSO HLA Typing Kit, Invitrogen). Genomic DNA was amplified using locus-specific primers flanking exons 2 and 3 of the HLA class I genes. The PCR products were hybridized to an array of immobilized sequence-specific oligonucleotide probes. The probe-bound amplified product was detected by a color formation assay. All assays were automated using the AutoRELI 48 Instrument (Dynal Biotech). The HLA-B alleles were grouped into HLA bw4 and HLA bw6 epitopes according to the official page of HLA nomenclature [30].

### Statistical analysis

Genotype frequency comparisons between groups were performed by two-tailed Fisher's exact test in R package for each SNP (p-values of 2x3 tables). The frequency of HLA alleles was also analyzed by two-tailed Fisher's exact test in R package (p-values of 2x2 tables). The results were corrected for multiple hypothesis testing to control the Benjamini–Hochberg false discovery rate (FDR) at a significant threshold of 0.1 to compare LTNP with different control populations (q-value). A similar correction was made to compare different subcategories of LTNP individuals with control populations, using a significant threshold of 0.05 (q-value).

## Results

### SNP and polymorphisms associated with the *Spanish long term non progressors cohort* phenotype

The individuals included in the analysis were genotyped for 14 different SNP and the *CCR5*-Δ32 polymorphism. Eleven out of 14 SNP did not differ significantly between LTNP and groups of healthy donors, AIDS patients and typical progressors (Table 2).

**Table 2:** Genotype distribution of different single nucleotide polymorphisms in distinct groups of HIV patients and in healthy donors.

*SNP*	*Group (n)*	*Genotype distribution*						*p-value*^a^	*FDR*^b^
		*n*	*%*	*n*	*%*	*n*	*%*		
***CCR5–2459 (G/A)***		***GG***		***GA***		***AA***			
***rs1799987***	*HD (122)*	*25*	*20*.*5*	*53*	*43*.*4*	*44*	*36*.*1*	*0*.*0236*	*ns*
	*LTNP (127)*	*33*	*26*.*0*	*68*	*53*.*5*	*26*	*20*.*5*	-	-
***SDF-1 3'UTR 801 (G/A)***		***GG***		***GA***		***AA***			
***rs1801157***	*HD (158)*	*98*	*62*.*0*	*49*	*31*.*0*	*11*	*7*.*0*	*ns*	*ns*
	*LTNP (117)*	*73*	*62*.*4*	*38*	*32*.*5*	*6*	*5*.*1*	-	-
***RANTES -403 (G/A)***		***GG***		***GA***		***AA***			
***rs2107538***	*HD (164)*	*114*	*69*.*5*	*44*	*26*.*8*	*6*	*3*.*7*	*ns*	*ns*
	*AIDS (55)*	*34*	*61*.*8*	*18*	*32*.*7*	*3*	*5*.*5*	*ns*	*ns*
	*TP (212)*	*145*	*68*.*4*	*58*	*27*.*4*	*9*	*4*.*2*	*ns*	*ns*
	*LTNP (130)*	*90*	*69*.*2*	*37*	*28*.*5*	*3*	*2*.*3*	-	-
***CD32α +494 (A/G)***		***AA***		***AG***		***GG***			
***rs1801274***	*HD (159)*	*42*	*26*.*4*	*86*	*54*.*1*	*31*	*19*.*5*	*ns*	*ns*
	*AIDS (55)*	*12*	*21*.*8*	*31*	*56*.*4*	*12*	*21*.*8*	*ns*	*ns*
	*TP (213)*	*42*	*19*.*7*	*111*	*52*.*1*	*60*	*28*.*2*	*ns*	*ns*
	*LTNP (124)*	*32*	*25*.*8*	*61*	*49*.*2*	*31*	*25*.*0*	-	-
***Tsg101–517 (C/T)***		***CC***		***CT***		***TT***			
***rs1857909***	*HD (259)*	*209*	*80*.*7*	*49*	*18*.*9*	*1*	*0*.*4*	*ns*	*ns*
	*AIDS (55)*	*44*	*80*.*0*	*10*	*18*.*2*	*1*	*1*.*8*	*ns*	*ns*
	*TP (213)*	*177*	*83*.*1*	*35*	*16*.*4*	*1*	*0*.*5*	*ns*	*ns*
	*LTNP (130)*	*115*	*88*.*5*	*15*	*11*.*5*	*0*	*0*	-	-
***Rab27a 3'UTR (C/T)***		***CC***		***CT***		***TT***			
***rs1050931***	*HD (248)*	*161*	*64*.*9*	*76*	*30*.*6*	*11*	*4*.*4*	*ns*	*ns*
	*AIDS (54)*	*36*	*66*.*7*	*16*	*29*.*6*	*2*	*3*.*7*	*ns*	*ns*
	*TP (211)*	*144*	*68*.*2*	*57*	*27*.*0*	*10*	*4*.*7*	*ns*	*ns*
	*LTNP (130)*	*87*	*66*.*9*	*39*	*30*.*0*	*4*	*3*.*1*	-	-
***Rggta (G/A)***		***GG***		***GA***		***AA***			
***rs729421***	*HD (177)*	*68*	*38*.*4*	*89*	*50*.*3*	*20*	*11*.*3*	*ns*	*ns*
	*AIDS (55)*	*16*	*29*.*1*	*29*	*52*.*7*	*10*	*18*.*2*	*ns*	*ns*
	*TP (213)*	*80*	*37*.*6*	*102*	*47*.*9*	*31*	*14*.*6*	*ns*	*ns*
	*LTNP (111)*	*52*	*46*.*8*	*59*	*53*.*2*	*18*	*16*.*2*	-	-
***αCatenin 3'UTR (G/T)***		***GG***		***GT***		***TT***			
***rs288039***	*HD (163)*	*84*	*51*.*5*	*66*	*40*.*5*	*13*	*8*.*0*	*ns*	*ns*
	*AIDS (55)*	*26*	*47*.*3*	*22*	*40*.*0*	*7*	*12*.*7*	*ns*	*ns*
	*TP (212)*	*114*	*53*.*8*	*82*	*38*.*7*	*16*	*7*.*5*	*ns*	*ns*
	*LTNP (69)*	*40*	*58*.*0*	*23*	*33*.*3*	*6*	*8*.*7*	-	-
***αCatenin 3'UTR (A/T)***		***AA***		***AT***		***TT***			
***rs3749663***	*HD (260)*	*136*	*52*.*3*	*102*	*39*.*2*	*22*	*8*.*5*	*ns*	*ns*
	*AIDS (55)*	*25*	*45*.*5*	*23*	*41*.*8*	*7*	*12*.*7*	*ns*	*ns*
	*TP (212)*	*114*	*53*.*8*	*81*	*38*.*2*	*17*	*8*.*0*	*ns*	*ns*
	*LTNP (129)*	*71*	*55*.*0*	*47*	*36*.*4*	*11*	*8*.*5*	-	-
***αCatenin intron (C/T)***		***CC***		***CT***		***TT***			
***rs700626***	*HD (168)*	*92*	*54*.*8*	*66*	*39*.*3*	*10*	*6*.*0*	*ns*	*ns*
	*LTNP (69)*	*39*	*56*.*5*	*24*	*34*.*8*	*6*	*8*.*7*	-	-
***HCP5 3'UTR (T/G)***		***TT***		***TG***		***GG***			
***rs2395029***	*HD (254)*	*245*	*96*.*5*	*9*	*3*.*5*	*0*	*0*	***3*.*94x10***^***-8***^	***1*.*5x10***^***-6***^
	*AIDS (55)*	*51*	*92*.*7*	*4*	*7*.*3*	*0*	*0*	*0*.*0189*	*ns*
	*TP (213)*	*191*	*89*.*6*	*22*	*10*.*4*	*0*	*0*	***0*.*0044***	***0*.*057***
	*LTNP (128)*	*100*	*78*.*2*	*28*	*21*.*8*	*0*	*0*	-	-
***CCR2-V64I +190 (G/A)***		***GG***		***GA***		***AA***			
***rs1799864***	*HD (262)*	*220*	*84*.*0*	*40*	*15*.*3*	*2*	*0*.*8*	***0*.*0097***	***0*.*092***
	*AIDS (55)*	*44*	*80*.*0*	*10*	*18*.*2*	*1*	*1*.*8*	*ns*	*ns*
	*TP (212)*	*163*	*76*.*9*	*46*	*21*.*7*	*3*	*1*.*4*	*ns*	*ns*
	*LTNP (129)*	*92*	*71*.*3*	*34*	*26*.*4*	*3*	*2*.*3*	-	-
***CCR5 Δ32 (WT/Δ32)***		***WT/WT***		***WT/Δ32***		***Δ32/Δ32***			
***rs333***	*HD (246)*	*204*	*82*.*9*	*40*	*16*.*3*	*2*	*0*.*8*	*ns*	*ns*
	*AIDS (55)*	*50*	*90*.*9*	*5*	*9*.*1*	*0*	*0*	*0*.*0245*	*ns*
	*TP (213)*	*173*	*81*.*2*	*40*	*18*.*8*	*0*	*0*	*ns*	*ns*
	*LTNP (129)*	*98*	*76*	*31*	*24*.*0*	*0*	*0*	-	-
***5'HLA-C (C/T)***		***CC***		***CT***		***TT***			
***rs9264942***	*AIDS (55)*	*16*	*29*.*1*	*22*	*40*.*0*	*17*	*31*.*9*	*ns*	*ns*
	*TP (212)*	*41*	*19*.*3*	*104*	*49*.*1*	*67*	*31*.*6*	***0*.*0045***	***0*.*057***
	*LTNP (128)*	*36*	*28*.*1*	*71*	*55*.*5*	*21*	*16*.*4*	-	-
***IL-10–592 (C/A)***		***CC***		***CA***		***AA***			
***rs1800872***	*HD (250)*	*142*	*56*.*8*	*87*	*34*.*8*	*21*	*8*.*4*	*ns*	*ns*
	*AIDS (54)*	*30*	*55*.*6*	*17*	*31*.*5*	*7*	*13*.*0*	*ns*	*ns*
	*TP (208)*	*113*	*54*.*3*	*85*	*40*.*9*	*10*	*4*.*8*	*ns*	*ns*
	*LTNP (125)*	*71*	*56*.*8*	*49*	*39*.*2*	*5*	*4*.*0*	-	-

HD: healthy donors, TP: typical progressors, AIDS: HIV patients with AIDS, LTNP: long term non progressors.

^a^p-values were calculated for each SNP comparing the genotypes of LTNP population with the other groups using Fisher's exact test (p<0.05 were considered significant, ns, not significant).

^b^False discovery rate (FDR) correction for multiple testing (alpha = 0.1 were considered significant, ns, not significant).

However, a significant difference in the genotype distribution was identified in 3 SNP (*HCP5*, *CCR2* and 5’*HLA-C*) (Table 2). In the case of *HCP5*, a clearly higher frequency of the genotype TG was found in LTNP compared with HD and TP groups, and less significant with AIDS patients (Table 2). The differences in the GA/AA genotype distribution of the SNP causing the V64I mutation in *CCR2* (HIV-1 co-receptor that is associated with protection [3]) were highly significant when comparing LTNP with HD. Regarding the Δ32 deletion of the *CCR5* HIV-1 co-receptor locus that is associated with delayed HIV disease progression [1, 2, 5], a higher frequency of the protective WT/Δ32 genotype was observed in LTNP than in the AIDS group, but these differences did not reach statistical significance after FDR correction. The variant -35C/T located 35 kb upstream of the *HLA-C* locus has been associated with delayed HIV disease progression in infected patients [16]. Accordingly, a significantly higher frequency of the CC and CT genotypes was found in Spanish LTNP compared with TP (Table 2). Therefore, our data confirm the association of *HCP5*, *CCR2* and 5’*HLA-C* SNPs to LTNP phenotype.

### Genotype distribution of significant SNP and CCR5-Δ32 polymorphism in distinct subcategories of the *Spanish LTNP cohort*

**Fig 1:**
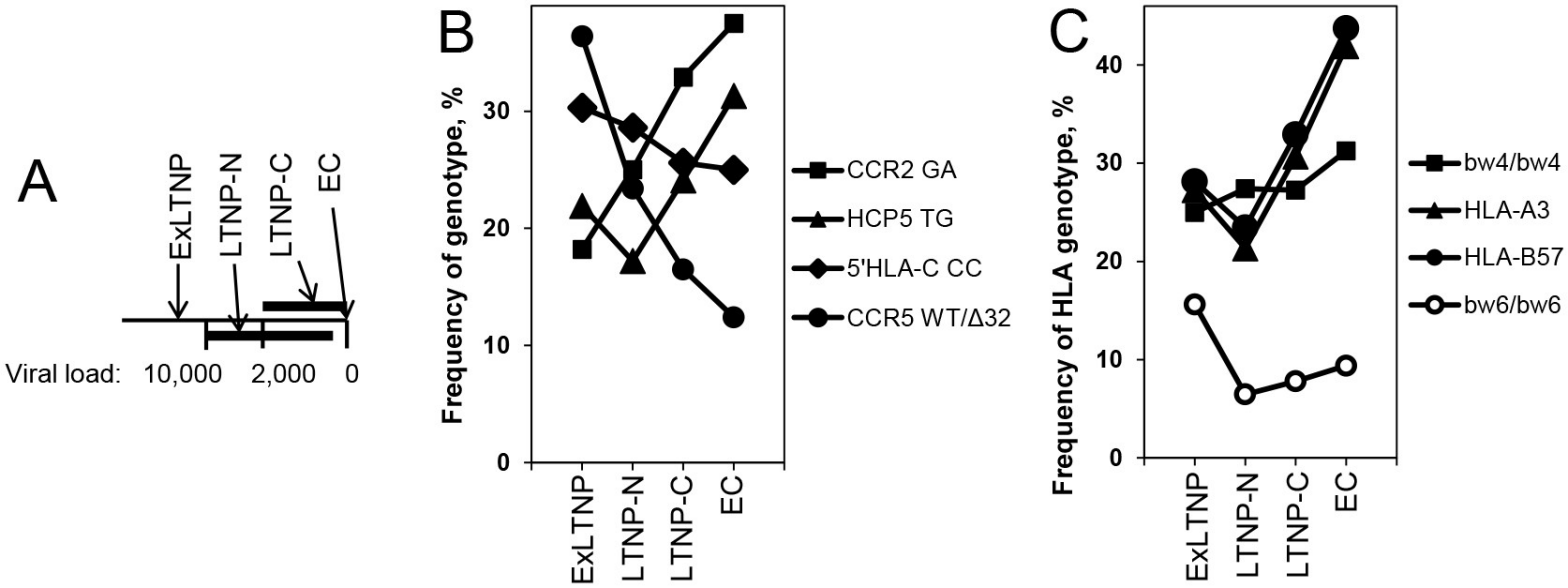
Genotype frequencies among subpopulations of LTNP as a function of viral load. The frequencies of the indicated SNP or *CCR5*-Δ32 genotypes (B) or HLA genotypes (C) relative to the total number of individuals are plotted for the LTNP subcategories that are graphically depicted (A): ExLTNP, patients who were LTNP for 10 years but thereafter failed to fulfill any of the inclusion criteria; LTNP-N, viremic non controller LTNP, VL>50–10,000; LTNP-C, controllers, VL<2,000 copies/mL; EC, elite controllers, undetectable VL. Displayed are relevant SNP and HLA genotypes with a frequency in the LTNP subcategories above 15%, indicating those that are more frequent (filled symbols) or less frequent (open symbols) in the overall LTNP population than in HD, according to Table 3 and Fig 2.

As described in Methods section LTNP were stratified according to VL into 4 subcategories, ExLTNP, viremic non controllers LTNP-N, controllers LTNP-C and elite controllers EC (Fig 1A), and the genotype frequencies of the relevant genetic factors were determined (i.e. *HCP5*, *CCR2* and 5’*HLA-C* SNPs). The results confirmed the protective nature of the *HCP5* and *CCR2* genotypes, as they were more frequent in most subcategories of LTNP, especially in those subcategories with the lowest VL, the LTNP-C and the EC, than in the other HIV-infected or HD populations (Table 3). For a summary and statistics see Table 4. Actually, *HCP5* and *CCR2* SNP frequencies were gradually increased within LTNP subcategories in an inverse correlation with VL (framed data in Table 3), with percentages of *HCP5* and *CCR2* favorable genotypes peaking at the EC population with undetectable VL (Fig 1B). For a summary and statistics see Table 4.

**Table 3:** Genotype distribution of selected SNP, which have specific alleles associated with protection or with disease progression, in distinct subcategories of HIV LTNP patients and in healthy donors.

*SNP*	*Group (n)*	*Genotype distribution*						*p-value*^a^			*FDR*^b^		
		*n*	*%*	*n*	*%*	*n*	*%*						
*HCP5 3'UTR (T/G)*		*TT*		*TG*		*GG*		*HD*	*AIDS*	*TP*	*HD*	*AIDS*	*TP*
***rs2395029***	*HD (254)*	*245*	*96*.*5*	*9*	*3*.*5*	*0*	*0*	-			-		
	*AIDS (55)*	*51*	*92*.*7*	*4*	*7*.*3*	*0*	*0*	*ns*	-		*ns*	-	
	*TP (213)*	*191*	*89*.*6*	*22*	*10*.*4*	*0*	*0*	***0*.*0045***	*ns*	-	***0*.*022***	*ns*	-
	*ExLTNP (32)*	*25*	*78*.*1*	*7*	*21*.*9*	*0*	*0*	***<10***^***−3***^	*ns*	*ns*	***0*.*005***	*ns*	*ns*
	*LTNP-N (64)*	*53*	*82*.*8*	*11*	*17*.*2*	*0*	*0*	***<10***^***−3***^	*ns*	*ns*	***0*.*005***	*ns*	*ns*
	*LTNP-C (79)*	*60*	*75*.*9*	*19*	*24*.*1*	*0*	*0*	***<10***^***−6***^	***0*.*0112***	***0*.*004***	***<10***^***−4***^	***0*.*044***	***0*.*022***
	*EC (32)*	*22*	*68*.*8*	*10*	*31*.*3*	*0*	*0*	***<10***^***−5***^	***<10***^***−2***^	***0*.*003***	***<10***^***−4***^	***0*.*024***	***0*.*02***
***CCR2-V64I (G/A)***		***GG***		***GA***		***AA***							
***rs1799864***	*HD (262)*	*220*	*84*.*0*	*40*	*15*.*3*	*2*	*0*.*8*	-			-		
	*AIDS (55)*	*44*	*80*.*0*	*10*	*18*.*2*	*1*	*1*.*8*	*ns*	-		*ns*	-	
	*TP (212)*	*163*	*76*.*9*	*46*	*21*.*7*	*3*	*1*.*4*	*ns*	*ns*	-	*ns*	*ns*	-
	*ExLTNP (33)*	*26*	*78*.*8*	*6*	*18*.*2*	*1*	*3*.*0*	*ns*	*ns*	*ns*	*ns*	*ns*	*ns*
	*LTNP-N (64)*	*46*	*71*.*9*	*16*	*25*.*0*	*2*	*3*.*1*	***0*.*0471***	*ns*	*ns*	*ns*	*ns*	*ns*
	*LTNP-C (79)*	*51*	*64*.*6*	*26*	*32*.*9*	*2*	*2*.*5*	***<10***^***−3***^	*ns*	*ns*	***0*.*005***	*ns*	*ns*
	*EC (32)*	*20*	*62*.*5*	*12*	*37*.*5*	*0*	*0*	***0*.*0203***	*ns*	*ns*	*0*.*066*	*ns*	*ns*
***5'HLA-C (C/T)***		***CC***		***CT***		***TT***							
***rs9264942***	*AIDS (55)*	*16*	*29*.*1*	*22*	*40*.*0*	*17*	*31*.*9*	-	-		-		
	*TP (212)*	*41*	*19*.*3*	*104*	*49*.*1*	*67*	*31*.*6*	-	*ns*	-	-	*ns*	
	*ExLTNP (33)*	*10*	*30*.*3*	*18*	*54*.*5*	*5*	*15*.*2*	-	*ns*	*ns*	-	*ns*	*ns*
	*LTNP-N (63)*	*18*	*28*.*6*	*36*	*57*.*1*	*9*	*14*.*3*	-	*ns*	***0*.*015***	-	*ns*	***0*.*054***
	*LTNP-C (78)*	*20*	*25*.*6*	*45*	*57*.*7*	*13*	*16*.*7*	-	*ns*	***0*.*033***	-	*ns*	*0*.*099*
	*EC (32)*	*8*	*25*.*0*	*17*	*53*.*1*	*7*	*21*.*9*	-	*ns*	*ns*	-	*ns*	*ns*

HD: healthy donors; TP: typical progressors; AIDS: HIV patients with AIDS; LTNP: long term non progressors; ExLTNP: patients who were LTNP for 10 years but thereafter failed to fulfill any of the inclusion criteria; LTNP-N: viremic LTNP with VL >10,000 copies/ml; LTNP-C: LTNP with VL <2,000 copies/ml; EC: elite controllers with undetectable VL.

^a^p-values were calculated for each SNP comparing the genotypes of LTNP population with the other groups using Fisher's exact test (p<0.05 were considered significant, ns, not significant).

^b^False discovery rate (FDR) correction for multiple testing (alpha = 0.1 were considered significant, ns, not significant).

**Table 4:** Summary and statistics for differences in frequencies of the 16 genotypes and alleles associated with protection or disease progression described in this report. Statistics apply to comparisons among distinct subcategories of Spanish LTNP patients, and with other groups of Spanish HIV patients and healthy donors.

	*SNP / HLA allele*	*Group*^a^	*p-value*^a^			*FDR*^b^		
			*HD*	*AIDS*	*TP*	*HD*	*AIDS*	*TP*
***P1***^a^	***HLA-B57***	*LTNP (128)*	***<10***^***−7***^	-	-	***<2*.*3x10***^***-6***^	-	-
		*ExLTNP (32)*	***1*.*0x10***^***-4***^	-	-	***<5*.*3x10***^***-4***^	-	-
		*LTNP-N (64)*	***1*.*0x10***^***-4***^	-	-	***<5*.*3x10***^***-4***^	-	-
		*LTNP-C (79)*	***1*.*0x10***^***-4***^	-	-	***<5*.*3x10***^***-4***^	-	-
		*EC (32)*	***1*.*0x10***^***-4***^	-	-	***<5*.*3x10***^***-4***^	-	-
***P2***	***HCP5 3'UTR***	*LTNP (128)*	***<10***^***−7***^	***2*.*0x10***^***-2***^	***4*.*0x10***^***-3***^	***<2*.*3x10***^***-6***^	***3*.*0x10***^***-2***^	***8*.*9x10***^***-3***^
	***rs2395029 (TG)***	*ExLTNP (32)*	***6*.*0x10***^***-4***^	*ns*	*ns*	***2*.*3x10***^***-3***^	*ns*	*ns*
		*LTNP-N (64)*	***4*.*0x10***^***-4***^	*ns*	*ns*	***1*.*6x10***^***-3***^	*ns*	*ns*
		*LTNP-C (79)*	***<10***^***−7***^	***1*.*0x10***^***-2***^	***4*.*0x10***^***-3***^	***<2*.*3x10***^***-6***^	***1*.*7x10***^***-2***^	***8*.*9x10***^***-3***^
		*EC (32)*	***<10***^***−5***^	***6*.*0x10***^***-3***^	***3*.*0x10***^***-3***^	***1*.*7x10***^***-4***^	***1*.*2x10***^***-2***^	***7*.*4x10***^***-3***^
***P3***	***HLA bw4/bw4***	*LTNP (128)*	***1*.*0x10***^***-4***^	-	-	***<5*.*3x10***^***-4***^	-	-
		*ExLTNP (32)*	***4*.*9x10***^***-2***^	-	-	*ns*	-	-
		*LTNP-N (64)*	***1*.*6x10***^***-3***^	-	-	***5*.*5x10***^***-3***^	-	-
		*LTNP-C (79)*	***1*.*0x10***^***-4***^	-	-	***<5*.*3x10***^***-4***^	-	-
		*EC (32)*	***1*.*7x10***^***-3***^	-	-	***5*.*6x10***^***-3***^	-	-
***P4***	***HLA-B52***	*LTNP (128)*	***2*.*0x10***^***-3***^	-	-	***5*.*8x10***^***-3***^	-	-
		*ExLTNP (32)*	***1*.*0x10***^***-2***^	-	-	***1*.*7x10***^***-2***^	-	-
		*LTNP-N (64)*	***2*.*9x10***^***-2***^	-	-	***4*.*1x10***^***-2***^	-	-
		*LTNP-C (79)*	***2*.*1x10***^***-2***^	-	-	***3*.*1x10***^***-2***^	-	-
***P5***	***HLA-B27***	*LTNP (128)*	***2*.*0x10***^***-3***^	-	-	***5*.*8x10***^***-3***^	-	-
		*LTNP-N (64)*	***2*.*0x10***^***-4***^	-	-	***9*.*9x10***^***-4***^	-	-
		*LTNP-C (79)*	***1*.*5x10***^***-2***^	-	-	***2*.*4x10***^***-2***^	-	-
***P6***	***CCR2-V64I***	*LTNP (129)*	***5*.*0x10***^***-3***^	*ns*	*ns*	***1*.*1x10***^***-2***^	*ns*	*ns*
	***rs1799864 (GA/AA)***	*LTNP-N (64)*	***3*.*0x10***^***-2***^	*ns*	*ns*	***4*.*1x10***^***-2***^	*ns*	*ns*
		*LTNP-C (79)*	***4*.*0x10***^***-4***^	*ns*	***4*.*0x10***^***-2***^	***1*.*6x10***^***-3***^	*ns*	***5*.*0x10***^***-2***^
		*EC (32)*	***6*.*0x10***^***-3***^	*ns*	*ns*	***1*.*2x10***^***-2***^	*ns*	*ns*
***P7***	***5'HLA-C***	*LTNP (128)*	-	***3*.*0x10***^***-2***^	***2*.*0x10***^***-3***^	-	***4*.*1x10***^***-2***^	***5*.*8x10***^***-3***^
	***rs9264942 (CC/CT)***	*LTNP-N (63)*	-	***4*.*0x10***^***-2***^	***6*.*0x10***^***-3***^	-	***5*.*0x10***^***-2***^	***1*.*2x10***^***-2***^
		*LTNP-C (78)*	-	*ns*	***1*.*0x10***^***-2***^	-	*ns*	***1*.*7x10***^***-2***^
***P8***	***HLA-A03***	*LTNP (125)*	***1*.*0x10***^***-2***^	-	-	***1*.*7x10***^***-2***^	-	-
		*LTNP-C (75)*	***1*.*0x10***^***-2***^	-	-	***1*.*7x10***^***-2***^	-	-
		*EC (31)*	***3*.*0x10***^***-3***^	-	-	***7*.*4x10***^***-3***^	-	-
***P9***	***HLA-B39*** ^c^	*LTNP (128)*	***2*.*0x10***^***-2***^	-	-	***3*.*0x10***^***-2***^	-	-
		*ExLTNP (32)*	***1*.*0x10***^***-2***^	-	-	***1*.*7x10***^***-2***^	-	-
***R1a***	***HLA bw6/bw6***	*LTNP (128)*	***1*.*0x10***^***-4***^	-	-	***<5*.*3x10***^***-4***^	-	-
		*LTNP-N (64)*	***1*.*0x10***^***-4***^	-	-	***5*.*3x10***^***-4***^	-	-
		*LTNP-C (79)*	***1*.*0x10***^***-4***^	-	-	***<5*.*3x10***^***-4***^	-	-
		*EC (32)*	***3*.*4x10***^***-2***^	-	-	***4*.*5x10***^***-2***^	-	-
***R2***	***HLA-B18***	*LTNP (128)*	***9*.*0x10***^***-4***^	-	-	***3*.*3x10***^***-3***^	-	-
		*LTNP-N (64)*	***4*.*7x10***^***-2***^	-	-	*ns*	-	-
		*LTNP-C (79)*	***3*.*0x10***^***-4***^	-	-	***1*.*4x10***^***-3***^	-	-
		*EC (32)*	***3*.*7x10***^***-2***^	-	-	***4*.*8x10***^***-2***^	-	-
***R3***	***HLA-A24***	*LTNP (125)*	***2*.*5x10***^***-3***^	-	-	***6*.*9x10***^***-3***^	-	-
		*LTNP-N (61)*	***1*.*3x10***^***-2***^	-	-	***2*.*1x10***^***-2***^	-	-
		*LTNP-C (75)*	***2*.*6x10***^***-3***^	-	-	***6*.*9x10***^***-3***^	-	-
***R4***	***HLA-B08***	*LTNP (128)*	***3*.*6x10***^***-3***^	-	-	***8*.*6x10***^***-3***^	-	-
		*LTNP-C (79)*	***2*.*4x10***^***-2***^	-	-	***3*.*5x10***^***-2***^	-	-
***R5***	***HLA-A29***	*LTNP (125)*	***1*.*1x10***^***-2***^	-	-	***1*.*8x10***^***-2***^	-	-

^a^Genotypes or alleles are labelled P for ‘protection’ or R for ‘risk’ depending on whether their frequency is higher or lower in the indicated LTNP population. respectively. and roughly numerically ordered from most protective and with the highest statistical power. P1. and from most risky and with the highest statistical significance. R1.

^b^ Only statistics for significant differences are listed (p<0.05); ns, not significant.

^c^ Novel genetic factors described in this report in association with LTNP condition are framed.

The enrichment of the *HCP5* and *CCR2* favorable genotypes in EC-LTNP with undetectable VL was somehow expected. However, it is very noticeable that the LTNP-C controllers, whose VL are always maintained below 2,000 copies/mL, are also very significantly endowed with these protective genotypes (p-values in Table 3). This suggests that viral replication limited to this threshold value for many years may also be a marker of a profound and stable LTNP status.

### Allelic frequencies of HLA-A and -B in the *Spanish LTNP cohort*

**Fig 2:**
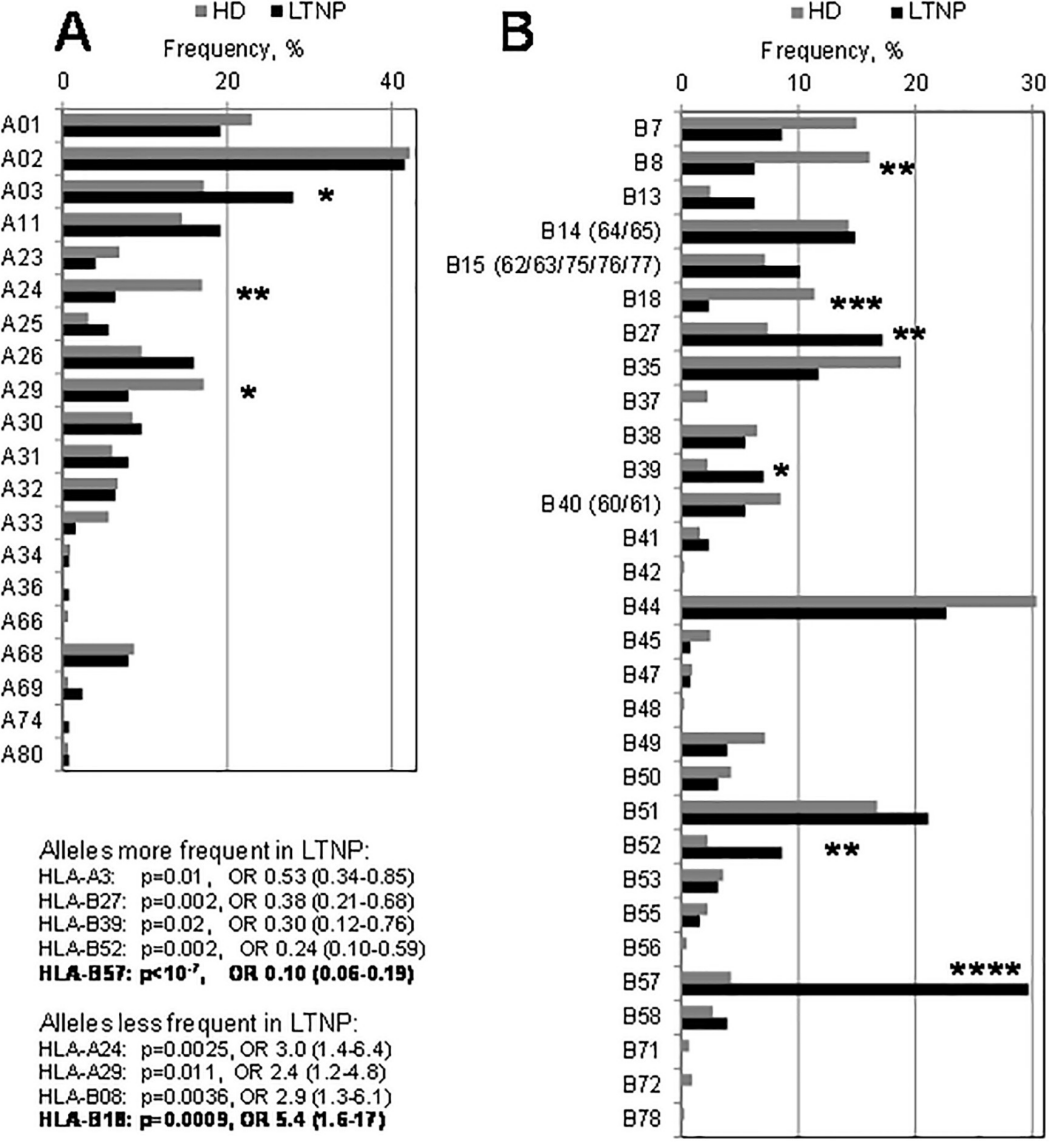
Comparison of the frequencies of HLA-A and HLA-B individuals between LTNP and healthy donor populations. HD, n = 448 donors for HLA-A and HLA-B; LTNP, n = 125 patients for HLA-A and 128 patients for HLA-B. Statistically significant differences are marked with asterisks (*, p<0.05; **, p<0.01; ****, p<0.0001) and detailed in the text inset. Relevant frequencies for HD vs. LTNP are: HLA-A03 (17 vs. 28%), HLA-A24 (17 vs 6.4%), HLA-A29 (17 vs. 8.0%), HLA-B8 (16 vs. 6.3%), HLA-B18 (11 vs 2.3%), HLA-B27 (7.4 vs. 17%), HLA-B39 (2.2 vs. 7.0%), HLA-B52 (2.2 vs. 8.6%) and HLA-B57 (4.2 vs. 30%).

LTNP and HD were typed for HLA class I. Most HLA alleles were not significantly different between the LTNP and the control group. From those with significant differences, several alleles seemed to favor the LTNP condition, as their allelic frequencies were significantly higher in LTNP than in HD (Fig 2); these included HLA-B57, followed by HLA-B27, -B52, -A03 and -B39. In contrast, HLA-B18 was markedly less frequent in the LTNP population, as well as HLA-A24, -B08 and -A29, and thus appeared to be detrimental for LTNP status. Stratification of the LTNP into subcategories was undertaken for most relevant alleles. Given the high number of alleles for these two HLA loci, a very low number of patients was left in most subcategories and precluded statistical analysis. Still, the strongest favorable factor HLA-B57, together with -B52, -B27 and -A03, as well as the strongest unfavorable factor HLA-B18, together with -A24 and -B08, were significantly enriched in LTNP subcategories (Table 4). Interestingly, when the frequency of the HLA allele in the LTNP subcategories was above 10% and amenable to analysis, it showed again an inverse correlation of HLA-B57 and HLA-A03 protective alleles with VL (Fig 1C), as was the case for the favorable *HCP5* and *CCR2* SNP.

When the HLA-B alleles were classified according to their mutually exclusive bw4 or bw6 public epitopes [30], a highly significantly greater percentage of bw4 in homozygosity was observed in the LTNP compared with HD (Table 5), confirming these alleles as protective factors for the LTNP status. The converse association of bw6/bw6 homozygosity with risk for the LTNP condition was also as strong, and both extended to most LTNP subcategories (Tables 4 and 5). As before, favorable bw4/bw4 showed a mild inverse correlation with VL while unfavorable bw6/bw6 genotype showed a mild direct correlation with VL within Spanish LTNP subcategories (Fig 1C).

**Table 5:** Frequency comparison of HLA bw4 and bw6 allele groups between HD and LTNP subcategories.

	*Group (n)*	*Genotype distribution*						*p-values*^a^		*FDR*^b^	
		*n*	*%*	*n*	*%*	*n*	*%*	*bw4/bw4*	*bw6/bw6*	*bw4/bw4*	*bw6/bw6*
*HLA-B*		*bw4/bw4*		*bw4/bw6*		*bw6/bw6*					
	*HD (421)*	*69*	*16*.*4*	*239*	*56*.*8*	*113*	*26*.*8*	-	-	-	-
	*LTNP (128)*	*45*	*35*.*2*	*71*	*55*.*5*	*12*	*9*.*4*	*1*.*1x10*^*-5*^	***2*.*0x10***^***-5***^	*1*.*2x10*^*-4*^	***1*.*0x10***^***-4***^
	*ExLTNP (32)*	*10*	*31*.*3*	*17*	*53*.*1*	*5*	*15*.*6*	*0*.*049*	*ns*	*ns*	*ns*
	*LTNP-N (64)*	*22*	*34*.*4*	*38*	*59*.*4*	*4*	*6*.*3*	*0*.*002*	***1*.*2x10***^***-4***^	*0*.*003*	***2*.*4x10***^***-4***^
	*LTNP-C (79)*	*29*	*36*.*7*	*44*	*55*.*7*	*6*	*7*.*6*	*8*.*8x10*^*-5*^	***8*.*4x10***^***-5***^	*2*.*1x10*^*-4*^	***2*.*2x10***^***-4***^
	*EC (32)*	*13*	*40*.*6*	*16*	*50*	*3*	*9*.*4*	*0*.*002*	*0*.*034*	*0*.*003*	*0*.*042*

HD: healthy donors; TP: typical progressors; AIDS: HIV patients with AIDS; LTNP: long term non progressors; ExLTNP: patients who were LTNP for 10 years but thereafter failed to fulfill any of the inclusion criteria; LTNP-N: viremic LTNP with VL >10.000 copies/ml; LTNP-C: LTNP with VL <2.000 copies/ml; EC: elite controllers with undetectable VL.

^a^The presence of bw4/bw4 or bw6/bw6 genotypes in different categories of LTNP subcategories compared with healthy donors (HD) was calculated using Fisher's exact test (p<0.05 were considered statistically significant; ns. not significant)

^b^False discovery rate (FDR) correction for multiple testing (alpha = 0.1 were considered significant. ns. not significant).

### Overview of genetics and LTNP status in the *Spanish HIV cohorts*

**Fig 3:**
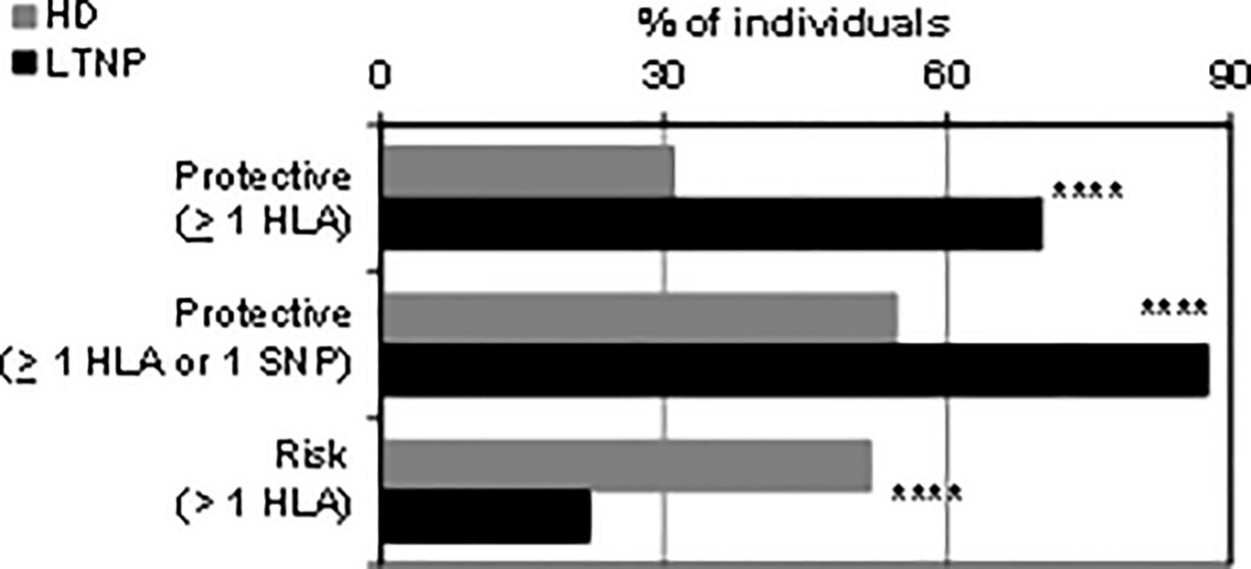
Individuals with a minimum of one protective or one risk factor for maintaining LTNP status: Frequency comparison between HD and LTNP. From top to bottom, the percentage of healthy controls and LTNP patients is indicated that have at least one protective HLA allele, at least one protective HLA allele or SNP factor, or at least one HLA risk allele, as listed in Table 4. ****, p< 0.0001. OR (95% confidence interval): 0.19 (0.12–0.30); 0.17 (0.09–0.31); 3.8 (2.4–6.1), respectively, from top to bottom. Stratification into LTNP subcategories did not provide additional information, as they were very homogeneous; p value was also <0.0001 for all comparisons between HD and each subcategory.

The 9 genotypes and alleles that are associated with LTNP status as well as those 5 unfavorable ones are listed in Table 4 and roughly ranked according to the intensity of the effect and the statistical significance. Interestingly, when analyzed as individuals concerning protective and risk factors, Spanish LTNP patients clearly stood up in comparison with HD. Almost 70% of LTNP patients had at least one HLA protective allele, and this rose to 87% when protective SNP were also considered. In contrast, only 22% of the LTNP had a detrimental allele (Fig 3). Fractions of HD controls having protective or risk alleles were very similar, for reference.

Notably, the mean number of protective minus risk HLA alleles and SNPs in individual Spanish healthy donors was balanced (0.74 protective– 0.62 risk to give a 0.12 balance per person, or an average of 0.12 protective HLA alleles and SNPs per healthy person). In sharp contrast, the mean was 12 times more marked for individual Spanish LTNP patients (1.66 protective– 0.23 risk to give an average of 1.43 protective HLA alleles and SNPs per LTNP patient), clearly indicating that LTNP is a population that has successfully undergone selection under the selective pressure of the HIV epidemics.

## Discussion

Several host genetic factors have been associated with HIV-1 disease progression in different cohorts of LTNP, typical progressors or rapid progressors, when compared with HIV seronegative individuals [1–25]. The present study aims to investigate the role of genetic factors in a large (n = 130) Spanish cohort of LTNP. However, the LTNP are a heterogeneous population consisting of HIV-1 infected individuals showing different phenotypes regarding their capacity to control viral replication. In this regard, the analysis has been extended to a conscientious stratification of LTNP, according to their VL, into elite controllers (EC), controllers (LTNP-C), viremic non-controller LTNP (LTNP-N) and individuals losing the LTNP status over time (ExLTNP).

Our analysis of the Spanish HIV-1 LTNP cohort and control healthy and infected populations, altogether representing 846 individuals, reveals 14 significant genetic factors. Nine of them are more frequent in the LTNP population, and thus qualify as factors that contribute to disease control and to LTNP status; in rough order of decreasing protective potency and statistical power these are the following alleles or genotypes: HLA-B57, *HCP5* TG rs2395029 SNP, HLA bw4/bw4 (p<0.0001, see individual details and summary in Table 4), HLA-B52, HLA-B27, *CCR2* GA/AA rs1799864 SNP (p<0.01,), *5’HLA-C* CC/CT rs9264942 SNP, HLA-A03 and HLA-B39 (0.01<p<0.05). Protective alleles/genotypes range each in frequency among the LTNP population from 7% to 30%, supporting the notion that a large proportion of the LTNP phenotype may be determined by accumulation of favorable genetic traits, rather by a single strongly protective factor. Conversely, 5 genetic factors are less frequently found in LTNP and appear to represent factors favoring disease progression; in rough order of decreasing risk and statistical power these are the following alleles or genotypes: HLA bw6/bw6, HLA-B18 (p<0.001), HLA-A24, HLA-B08, (p<0.01) and HLA-A29(p<0.05).

Two out of the 14 factors reported in Table 4 are described for the first time to our knowledge in firm association with any type of HIV susceptibility to infection or disease progression and, specifically, in association with the LTNP condition. Both are unfavorable HLA alleles, HLA-B08 and -A29. In addition, another 3 factors that have been found associated with other HIV conditions are described here for the first time in association with LTNP, including the protective HLA-B39 and the risk factors HLA-B18 and -A24. Furthermore, the positive association with LTNP of two more alleles, HLA-A03 and HLA-B52, for which very limited evidence is published, is confirmed with the Spanish LTNP cohort. In the natural history of HIV infection, several HLA class I alleles have been associated consistently with HIV progression, especially HLA-B alleles [10, 12, 14, 22, 31–34], and notably, we identify here novel HLA-B as well as HLA-A alleles. Identification of several new genetic associations when compared with studies on a geographically close population as the French cohort [35] stresses the importance of assembling and studying such cohorts of patients that control HIV infection, in spite of the scarcity of such patients. It also reveals the importance of thorough studies on novel cohorts such as the Spanish one reported here. Among other factors, one possible reason for the novelty of our data may relate to the high proportion of intravenous drug users among the Spanish LTNP compared with the majority of men who have sex with men (MSM) included in the French LTNP cohort.

The novel detrimental associations of HLA-B08 and HLA-A29 with maintenance of the LTNP status have little precedent in the literature. HLA-B08 within a common Western haplotype is frequently associated with fast progression of HIV disease, rapid CD4 T lymphocyte decline in adults and with increased mother to infant transmission [32,36,37], but the association has rarely been individually ascribed to HLA-B08. For HLA-A29 only a non-significant trend has been reported [38]. This negative association may interestingly be related to the poor recognition by A29-restricted T lymphocyte clones of viral sequence variants [39]. The large Spanish LTNP cohort data thus presents solid evidence for the first time on the negative association of these two HLA alleles with the LTNP status.

Concerning the three HLA alleles previously reported only in HIV patients other than LTNP, the effect of HLA-B39, which is identified here as a protective allele for the Spanish LTNP cohort, to our knowledge for the first time in association with LTNP, appears to depend on the study, the geographical area or the HIV-infected population. HLA-B39 was described as a risk allele in smaller populations of Argentinian HIV^+^ subjects [40] and of Indian serodiscordant couples [41], while, more in line with our results, as an allele associated with lower VL in Zambian HIV infected patients [42]. The second allele associated in this cohort for the first time with LTNP, HLA-A24, is described here as an unfavorable allele for the LTNP condition, and it was also early associated with rapid CD4 T lymphocyte decline [36] and with susceptibility in adults [43], promoting selection of cytotoxic T-lymphocyte escape variants in Japan [44, 45]. Whether the detrimental role of HLA-A24 for LTNP described here in the Spanish population is related to T-lymphocyte escape also in LTNP patients is currently unknown and warrants investigation. Finally, HLA-B18 was the strongest and most significant detrimental factor for the Spanish LTNP population. This HLA allele has been widely studied in HIV infected populations other than LTNP, and its favorable [38, 41] or risk [40, 46] contribution to diverse aspects of HIV disease is variable and at least seems to depend on the virus clade.

Further, HLA-A03 has been described in one report in association with French LTNP [35]. This early observation of positive association of HLA-A03 with LTNP is now confirmed with our larger and stricter Spanish LTNP cohort. Otherwise, A03 has also occasionally been associated with populations of HIV-infected patients other than LTNP [37, 47]. As for HLA-A03, we also describe a significant association of the HLA-B52 allele with delayed disease progression in the Spanish cohort of LTNP patients, confirming an international HIV controllers study [22] and a single earlier report weakly associating HLA-B52 with non-progression in a small Brazilian cohort of HIV-1 infected individuals [48].

Out of the 14 factors identified here in positive or negative association with Spanish LTNP, the remaining 7 factors were previously established, and our data are confirmatory. Previous studies have associated low HIV-1 viremia and prolonged survival with HLA-B57 [7, 10] and HLA-B27 [35] in HIV LTNP patients, and it is assumed that this is due to the antigen presentation by these alleles of conserved viral epitopes contributing to viral fitness. LTNP are also characterized by the SNP rs2395029 located at *HCP5* [18–21, 23], which is in tight linkage disequilibrium with the HLA-B*5701 allele [13]. The fact that these HLA-B alleles display the public HLA epitope bw4 is thought to underlie the previously described and here confirmed positive role of bw4/bw4 homozygosity [14] and the converse negative role of the bw6/bw6 genotype. Interestingly, when considering HLA supertypes [49], the LTNP-associated protective HLA alleles described here clustered together in some HLA supertypes (A03, B7, B27, B58 and B62 supertypes), and segregated away from the supertypes of risk alleles (A1, A24 and B44 supertypes). As the supertypes are based on HLA antigen presentation function to cytotoxic CD8^+^ T lymphocytes, this could possibly underlie the functional mechanism for their selective association in HIV-1 infection.

The present study confirms the strong protective effect for Spanish LTNP of *HCP5* 3’UTR TG rs2395029, *CCR2* GA/AA rs1799864 and *5’HLA-C* CC/CT rs9264942 SNPs.

When LTNP were stratified, gradual increases of the frequencies of favorable *HCP5*, HLA-B57, HLA-A03, *CCR2* and bw4/bw4 alleles and genotypes were concomitantly observed with increasing HIV-1 control capacity, peaking at LTNP-C and EC populations, confirming a trend previously assumed for some of them in other studies that analyzed a very limited number of LTNP patients [50]. Conversely, the strongly unfavorable bw6/bw6 genotype shows a mild inverse correlation with control of VL. However, this study shows that there is no such correlation of low VL with protective *5’HLA-C*, as published [51], nor with CCR5 Δ32 deletion, and questions including these two SNP as markers for reduced VL [50]. While the *CCR5 Δ32* deletion has extensively been confirmed to contribute to preventing initial HIV infection [1], these data may suggest that, once infection is established in patients, it does not contribute to maintaining a profound LTNP status as strongly as HCP5, HLA-B57, -A03, CCR2, or bw4/bw4 genotypes may do.

The classification of HIV-1 infected patients based on clinical data includes LTNP, typical progressors and rapid progressors. However, this classification can be enriched incorporating the VL measurement to define a more realistic description of the LTNP status with the subcategories included in the present study, i.e. EC-LTNP, LTNP-C, LTNP-N and ExLTNP. The genetic factors influencing the LTNP status have widely been studied, even from a genome-wide perspective [18–21]. However, the control of HIV-1 replication and the delayed disease progression simultaneously observed in EC-LTNP and LTNP-C have been poorly characterized. In this regard, the present study provides new clues about the effect of known factors influencing control and resistance to HIV-1 such as *HCP5*, *CCR2*, HLA-B57 and -A03 in EC-LTNP and LTNP-C compared with the rest of LTNP. On the other hand, well-documented genetic factors associated to LTNP status such as *CCR5* rs333 or 5’*HLA-C* do not seem to have any additive effect in the EC-LTNP or LTNP-C condition with respect to the rest of LTNP. Further studies are required to discern whether the EC-LTNP and LTNP-C statuses can be considered as an accumulation of several factors previously associated with EC or LTNP or as the presence of specific unknown associations with the simultaneous observation of both phenotypes.

The fact that with new cohorts like the large multicentric and stratified Spanish ones it is still possible to identify significant associations of the LTNP with 5 new HLA alleles (one protective and 4 detrimental for the LTNP condition) underscores the strong influence of HLA on viral control. It is still open whether especially the most significant unfavorable HLA-B18 allele could play a direct functional effect on control of HIV and in long-term stability of infected LTNP patients.

## Supporting information

S1 Table Primers and probes employed in the determination of rs333 and rs1801157. (DOCX)

## Abbreviations

AIDS: HIV-1 infected patients that have developed AIDS
CTL: cytotoxic T lymphocytes
EC-LTNP: Elite controller LTNP
ExLTNP: former LTNP
HD: healthy donors
LTNP: HIV-1 infected long-term non progressors
LTNP-C: controller LTNP
LTNP-N: viremic non controller LTNP
OR: odds ratio
SNP: single nucleotide polymorphisms
SSO: sequence-specific oligonucleotide
TP: HIV-1 infected typical progressors
VL: viral loads

## References

[1] Samson M, Libert F, Doranz BJ, et al Resistance to HIV-1 infection in caucasian individuals bearing mutant alleles of the CCR-5 chemokine receptor gene. Nature 1996;382:722–5. 10.1038/382722a0 8751444

[2] Dean M, Carrington M, Winkler C, et al Genetic restriction of HIV-1 infection and progression to AIDS by a deletion allele of the CKR5 structural gene. Hemophilia Growth and Development Study, Multicenter AIDS Cohort Study, Multicenter Hemophilia Cohort Study, San Francisco City Cohort, ALIVE Study. Science 1996;273:1856–62. 10.1126/science.273.5283.1856 8791590

[3] Smith MW, Dean M, Carrington M, et al Contrasting genetic influence of CCR2 and CCR5 variants on HIV-1 infection and disease progression. Hemophilia Growth and Development Study (HGDS), Multicenter AIDS Cohort Study (MACS), Multicenter Hemophilia Cohort Study (MHCS), San Francisco City Cohort (SFCC), ALIVE Study. Science 1997;277:959–65. 10.1126/science.277.5328.959 9252328

[4] Winkler C, Modi W, Smith MW, et al Genetic restriction of AIDS pathogenesis by an SDF-1 chemokine gene variant. Science 1998;279:389–93. 10.1126/science.279.5349.389 9430590

[5] Liu H, Chao D, Nakayama EE, et al Polymorphism in RANTES chemokine promoter affects HIV-1 disease progression. Proc Natl Acad Sci U S A 1999;96:4581–5. 10.1073/pnas.96.8.4581 10200305 PMC16375

[6] Shin HD, Winkler C, Stephens JC, et al Genetic restriction of HIV-1 pathogenesis to AIDS by promoter alleles of IL10. Proc Natl Acad Sci U S A 2000;97:14467–72. 10.1073/pnas.97.26.14467 11121048 PMC18942

[7] Carrington M, O'Brien SJ. The influence of HLA genotype on AIDS. Annu Rev Med 2003;54:535–51. 10.1146/annurev.med.54.101601.152346 12525683

[8] Machmach K, Abad-Molina C, Romero-Sanchez MC, et al IL28B single-nucleotide polymorphism rs12979860 is associated with spontaneous HIV control in white subjects. J Infect Dis 2013;207:651–5. 10.1093/infdis/jis717 23225905

[9] Ghezzi S, Galli L, Kajaste-Rudnitski A, et al Identification of TRIM22 single nucleotide polymorphisms associated with loss of inhibition of HIV-1 transcription and advanced HIV-1 disease. AIDS 2013;27:2335–44. 10.1097/01.aids.0000432474.76873.5f 23921607

[10] Migueles SA, Sabbaghian MS, Shupert WL, et al HLA B*5701 is highly associated with restriction of virus replication in a subgroup of HIV-infected long term nonprogressors. Proc Natl Acad Sci U S A 2000;97:2709–14. 10.1073/pnas.050567397 10694578 PMC15994

[11] Navis M, Schellens I, van BD, et al Viral replication capacity as a correlate of HLA B57/B5801-associated nonprogressive HIV-1 infection. J Immunol 2007;179:3133–43. 10.4049/jimmunol.179.5.3133 17709528

[12] Carrington M, Nelson GW, Martin MP, et al HLA and HIV-1: Heterozygote advantage and B*35-Cw*04 disadvantage. Science 1999;283:1748–52. 10.1126/science.283.5408.1748 10073943

[13] Gao X, Bashirova A, Iversen AK, et al AIDS restriction HLA allotypes target distinct intervals of HIV-1 pathogenesis. Nat Med 2005;11:1290–2. 10.1038/nm1333 16288280

[14] Flores-Villanueva PO, Yunis EJ, Delgado JC, et al Control of HIV-1 viremia and protection from AIDS are associated with HLA-Bw4 homozygosity. Proc Natl Acad Sci U S A 2001;98:5140–5. 10.1073/pnas.071548198 11309482 PMC33177

[15] Van Manen D, Kootstra NA, Boeser-Nunnink B, et al Association of HLA-C and HCP5 gene regions with the clinical course of HIV-1 infection. AIDS 2009;23:19–28. 10.1097/QAD.0b013e32831db247 19050382

[16] Thomas R, Apps R, Qi Y, et al HLA-C cell surface expression and control of HIV/AIDS correlate with a variant upstream of HLA-C. Nat Genet 2009;41:1290–4. 10.1038/ng.486 19935663 PMC2887091

[17] de Bakker PI, McVean G, Sabeti PC, et al A high-resolution HLA and SNP haplotype map for disease association studies in the extended human MHC. Nat Genet 2006;38:1166–72. 10.1038/ng1885 16998491 PMC2670196

[18] Dalmasso C, Carpentier W, Meyer L, et al Distinct genetic loci control plasma HIV-RNA and cellular HIV-DNA levels in HIV-1 infection: the ANRS Genome Wide Association 01 study. PLoS ONE 2008;3:e3907 10.1371/journal.pone.0003907 19107206 PMC2603319

[19] Le Clerc S, Limou S, Coulonges C, et al Genomewide association study of a rapid progression cohort identifies new susceptibility alleles for AIDS (ANRS Genomewide Association Study 03). J Infect Dis 2009;200:1194–201. 10.1086/605892 19754311

[20] Fellay J, Shianna KV, Ge D, et al A whole-genome association study of major determinants for host control of HIV-1. Science 2007;317:944–7. 10.1126/science.1143767 17641165 PMC1991296

[21] Limou S, Le CS, Coulonges C, et al Genomewide association study of an AIDS-nonprogression cohort emphasizes the role played by HLA genes (ANRS Genomewide Association Study 02). J Infect Dis 2009;199:419–26. 10.1086/596067 19115949

[22] Pereyra F, Jia X, McLaren PJ, et al The major genetic determinants of HIV-1 control affect HLA class I peptide presentation. Science 2010;330:1551–7. 10.1126/science.1195271 21051598 PMC3235490

[23] Guergnon J, Dalmasso C, Broet P, et al Single-nucleotide polymorphism-defined class I and class III major histocompatibility complex genetic subregions contribute to natural long-term nonprogression in HIV infection. J Infect Dis 2012;205:718–24. 10.1093/infdis/jir833 22238471

[24] McLaren PJ, Coulonges C, Ripke S, et al Association study of common genetic variants and HIV-1 acquisition in 6,300 infected cases and 7,200 controls. PLoS Pathog 2013;9:e1003515 10.1371/journal.ppat.1003515 23935489 PMC3723635

[25] Bartha I, Carlson JM, Brumme CJ, et al A genome-to-genome analysis of associations between human genetic variation, HIV-1 sequence diversity, and viral control. Elife 2013;2:e01123 10.7554/eLife.01123 24171102 PMC3807812

[26] Balas A, Garcia-Sanchez F, Vicario JL. Allelic and haplotypic HLA frequency distribution in Spanish hematopoietic patients. Implications for unrelated donor searching. Tissue Antigens 2011;77:45–53. 10.1111/j.1399-0039.2010.01578.x 21155721

[27] Garcia-Merino I, de Las CN, Jimenez JL, et al The Spanish HIV BioBank: a model of cooperative HIV research. Retrovirology 2009;6:27 10.1186/1742-4690-6-27 19272145 PMC2667474

[28] Caro-Murillo AM, Castilla J, Perez-Hoyos S, et al Spanish cohort of naive HIV-infected patients (CoRIS): rationale, organization and initial results. Enferm Infecc Microbiol Clin 2007;25:23–31. 17261243 10.1157/13096749

[29] Ballana E, Senserrich J, Pauls E, et al ZNRD1 (zinc ribbon domain-containing 1) is a host cellular factor that influences HIV-1 replication and disease progression. Clin Infect Dis 2010;50:1022–32. 10.1086/651114 20192730

[30] Robinson J, Halliwell JA, Hayhurst JD, et al The IPD and IMGT/HLA database: allele variant databases. Nucleic Acids Res 2015;43:D423–D431. 10.1093/nar/gku1161 25414341 PMC4383959

[31] Gao X, O'Brien TR, Welzel TM, et al HLA-B alleles associate consistently with HIV heterosexual transmission, viral load, and progression to AIDS, but not susceptibility to infection. AIDS 2010;24:1835–40. 10.1097/QAD.0b013e32833c3219 20588164 PMC2902625

[32] McNeil AJ, Yap PL, Gore SM, et al Association of HLA types A1-B8-DR3 and B27 with rapid and slow progression of HIV disease. QJM 1996;89:177–85. 10.1093/qjmed/89.3.177 8731561

[33] Yindom LM, Leligdowicz A, Martin MP, et al Influence of HLA class I and HLA-KIR compound genotypes on HIV-infection and markers of disease progression in a Manjako community in West Africa. J Virol 2010;84(16):8202–8. 10.1128/JVI.00116-10 20519398 PMC2916551

[34] Chikata T, Murakoshi H, Koyanagi M, et al Control of HIV-1 by an HLA-B*52:01-C*12:02 Protective Haplotype. J Infect Dis 2017;216(11):1415–1424. 10.1093/infdis/jix483 28968792

[35] Magierowska M, Theodorou I, Debre P, et al Combined genotypes of CCR5, CCR2, SDF1, and HLA genes can predict the long-term nonprogressor status in human immunodeficiency virus-1-infected individuals. Blood 1999;93:936–41. 9920843

[36] Kaslow RA, Duquesnoy R, VanRaden M, et al A1, Cw7, B8, DR3 HLA antigen combination associated with rapid decline of T-helper lymphocytes in HIV-1 infection. A report from the Multicenter AIDS Cohort Study. Lancet 1990;335:927–30. 10.1016/0140-6736(90)90995-h 1970024

[37] Kilpatrick DC, Hague RA, Yap PL, et al HLA antigen frequencies in children born to HIV-infected mothers. Dis Markers 1991;9:21–6. 1742942

[38] Farquhar C, Rowland-Jones S, Mbori-Ngacha D, et al Human leukocyte antigen (HLA) B*18 and protection against mother-to-child HIV type 1 transmission. AIDS Res Hum Retroviruses 2004;20:692–7. 10.1089/0889222041524616 15307911 PMC3380108

[39] Wilson CC, Kalams SA, Wilkes BM, et al Overlapping epitopes in human immunodeficiency virus type 1 gp120 presented by HLA A, B, and C molecules—Effects of viral variation on cytotoxic T-lymphocyte recognition. Journal of Virology 1997;71:1256–64. 8995649 10.1128/jvi.71.2.1256-1264.1997 PMC191180

[40] de Sorrentino AH, Marinic K, Motta P, et al HLA class I alleles associated with susceptibility or resistance to human immunodeficiency virus type 1 infection among a population in Chaco Province, Argentina. J Infect Dis 2000;182:1523–6. 10.1086/315854 11010837

[41] Chaudhari DV, Chavan VR, Ahir SP, et al Human leukocyte antigen B distribution in HIV discordant cohort from India. Immunol Lett 2013;156:1–6. 10.1016/j.imlet.2013.09.002 24029662

[42] Tang J, Tang S, Lobashevsky E, et al Favorable and unfavorable HLA class I alleles and haplotypes in Zambians predominantly infected with clade C human immunodeficiency virus type 1. J Virol 2002;76:8276–84. 10.1128/JVI.76.16.8276-8284.2002 12134033 PMC155130

[43] Keet IP, Tang J, Klein MR, et al Consistent associations of HLA class I and II and transporter gene products with progression of human immunodeficiency virus type 1 infection in homosexual men. J Infect Dis 1999;180:299–309. 10.1086/314862 10395843

[44] Goulder PJR, Edwards A, Phillips RE, et al Identification of a novel HLA-A24-restricted cytotoxic T-lymphocyte epitope within HIV-1 NEF. AIDS 1997;11:1883–4. 10.1097/00002030-199715000-00015 9412709

[45] Furutsuki T, Hosoya N, Kawana-Tachikawa A, et al Frequent transmission of cytotoxic-T-lymphocyte escape mutants of human immunodeficiency virus type 1 in the highly HLA-A24-positive Japanese population. J Virol 2004;78:8437–45. 10.1128/JVI.78.16.8437-8445.2004 15280452 PMC479048

[46] Leslie A, Matthews PC, Listgarten J, et al Additive contribution of HLA class I alleles in the immune control of HIV-1 infection. J Virol 2010;84:9879–88. 10.1128/JVI.00320-10 20660184 PMC2937780

[47] Zhang W, Wang L, Hong K, et al Frequency of HLA-A 03 associates with HIV-1 infection in a Chinese cohort. Sci China Life Sci 2013;56:1014–9. 10.1007/s11427-013-4555-4 24114445

[48] Teixeira SL, de Sa NB, Campos DP, et al Association of the HLA-B*52 allele with non-progression to AIDS in Brazilian HIV-1-infected individuals. Genes Immun 2014;15:256–62. 10.1038/gene.2014.14 24718028

[49] Sidney J, Peters B, Frahm N, et al HLA class I supertypes: a revised and updated classification. BMC Immunol 2008;9:1 10.1186/1471-2172-9-1 18211710 PMC2245908

[50] Casado C, Colombo S, Rauch A, et al Host and viral genetic correlates of clinical definitions of HIV-1 disease progression. PLoS ONE 2010;5:e11079 10.1371/journal.pone.0011079 20552027 PMC2884031

[51] Ballana E, Ruiz-de AA, Mothe B, et al Differential prevalence of the HLA-C -35 CC genotype among viremic long term non-progressor and elite controller HIV+ individuals. Immunobiology 2012;217:889–94. 10.1016/j.imbio.2011.12.012 22333575

