# Supplementary material for: Novel association of five HLA alleles with HIV-1 progression in Spanish long-term non progressor patients
Source: PLoS One. 2019 Aug 8;14(8):e0220459. doi: 10.1371/journal.pone.0220459 (PMC6687284; doi:10.1371/journal.pone.0220459)
Supplement: S1 Table — (DOCX) [file pone.0220459.s001.docx]

| **Gene** | **Polymorphism** | **Primer/probe** | **Sequence** |
| --- | --- | --- | --- |
| *CCR5* | rs333 | Forward | 5’AAGGTCTTCATTACACCTGCAGC3’ |
|  |  | Reverse | 5’AGCAGCGGCAGGACCA3’ |
|  |  | allele 1 | 5’FAM-ACAGTCAGTATCAATTCTGGAAGAATTTCCTA3’ |
|  |  | allele 2 | 5’VIC-TCTCATTTTCCATACATTAAAGATAGTCATCTTTA3’ |
| *SDF-1* | rs1801157 | Forward | 5’CGATCAACCTGGGCAAAGCC3’ |
|  |  | Reverse | 5’AGCTTTGGTCCTGAGAGTCC3’ |
|  |  | allele 1 | 5’FAM-TGGGAGCCGGGTCTGCCTCT3’ |
|  |  | allele 2 | 5’VIC-ACATGGGAGCCAGGTCTGCCTCTT3’ |

**SI Table. Primers and probes employed in the determination of rs333 and rs1801157.**
